# Timing of the First Dose of the Hepatitis B Vaccine in Preterm Infants

**DOI:** 10.3390/vaccines10101656

**Published:** 2022-10-02

**Authors:** Donna Lei, Taryn Miller, Jeremy Carr, Jim Buttery, Claudia A. Nold-Petry, Marcel F. Nold, Atul Malhotra

**Affiliations:** 1 Department of Paediatrics, Monash University, Melbourne, VIC 3168, Australia; 2 Monash Newborn, Monash Children’s Hospital, Melbourne, VIC 3168, Australia; 3 Department of Infection and Immunity, Monash Children’s Hospital, Melbourne, VIC 3168, Australia; 4 Centre for Health Analytics, Melbourne Children’s Campus, Melbourne, VIC 3052, Australia; 5 Department of Paediatrics, University of Melbourne, Melbourne, VIC 3052, Australia; 6 The Ritchie Centre, Hudson Institute of Medical Research, Melbourne, VIC 3168, Australia

**Keywords:** adverse effects, hepatitis B vaccination, immune response, preterm neonate, schedule

## Abstract

**Introduction:** The World Health Organization (WHO) recommends all newborn infants receive the first dose of the hepatitis B vaccine within 24 h of birth irrespective of maternal hepatitis B carrier status. However, the physiological immaturity of the immune system in preterm infants may influence the immune responses to the vaccine particularly in the first few days and weeks of life, and adverse events may occur following vaccination that are not observed in infants born at term. **Objectives:** To review existing published guidelines surrounding timing of the first dose of the hepatitis B vaccine in preterm infants born to hepatitis B surface antigen negative (HBsAg-negative) mothers. **Methods:** A search was performed for relevant papers and guidelines published between January 2002 and July 2022 on the Ovid MEDLINE and Embase databases and through targeted searches. Two authors independently reviewed the search results to identify relevant sources, which were then analysed and described through narrative synthesis. **Results:** Twenty-seven relevant papers and guidelines regarding 15 countries and regions were included. Of these, 13.3% of guidelines, which represented 16.8% of the overall population of 4.1 billion people covered by the identified guidelines, recommended a nationwide birth dose of the hepatitis B vaccine to all preterm infants. In 40.0% of guidelines (77.9% of the overall population), the birth dose was only recommended for infants with a birth weight of more than 2000–2200 g. Another 33.3% of countries and regions (covering 4.4% of the population) recommended no universal birth dose for all infants, including preterm infants, whilst 13.3% (1.0% of the population) had guidelines that varied between jurisdictions and hospitals within their country/region. **Conclusions:** Existing guidelines surrounding the timing of the first dose of the hepatitis B vaccine in preterm infants vary substantially between countries and regions. Further research comparing the immunogenicity and safety of different hepatitis B vaccine schedules is needed to provide concrete evidence to provide guidance regarding the timing of vaccination against hepatitis B in preterm infants.

## 1. Introduction

Hepatitis B is a viral infection of the liver caused by the hepatitis B virus (HBV) and remains a serious public health concern worldwide. One of the most common routes of transmission is vertically, i.e., through mother-to-child transmission at birth [1]. Perinatal hepatitis B transmission is estimated to account for up to 50% of hepatitis B carriers in countries such as China [2]. Compared to those infected with hepatitis B during adulthood, infants with hepatitis B are 90% more likely to develop chronic infection (overall 10–20% risk) therefore causing liver disease or cancer later in life [3]. The most effective way of preventing the spread of hepatitis B in general is through vaccination. Globally, hepatitis B immunisation has been effective in preventing vertical transmission [4]. The World Health Organization (WHO) thus recommends that all newborns, regardless of maternal hepatitis B surface antigen status, receive the first dose of the hepatitis B vaccine within 24 h of birth. This universal birth dose was recommended by WHO due to the failure of progression towards hepatitis B elimination goals using selective approaches to birth-dose immunisation based on maternal hepatitis B surface antigen (HBsAg) status. [5]. However, national action plans may differ, with low prevalence countries adopting a more targeted, screening-based vaccination policy. This review focuses on policies surrounding vaccination of preterm infants, and broader consideration of universal birth dose immunisation vs. risk-based approaches is beyond the scope of this review.

Limited research exists regarding optimal timing of the first dose of the hepatitis B vaccine specifically in preterm infants. Compared to term infants, premature neonates are at an increased risk of serious illness from diseases preventable by vaccines; besides hepatitis B, these include diphtheria, pertussis, and *Haemophilus influenzae* type B [6]. However, compared with term infants, the immunogenicity of hepatitis B vaccines is reduced in low birth weight and preterm infants [7]. This and other circumstances have led to discrepancies in practice worldwide, with some practitioners delaying the first dose of the hepatitis B vaccine. Further, recent research has shown that early administration of the hepatitis B vaccine (i.e., within the first 24 h of life) is associated with increased type 2 immune polarisation, which in turn was associated with cardiopulmonary disease in preterm neonates [8]. This review seeks to provide an overview of current practice, as informed by international guidelines around the timing of hepatitis B vaccination in preterm infants and the current criteria used to guide delayed administration of the first dose of the vaccine to hepatitis B surface antigen negative (HBsAg-negative) mothers in various countries and regions.

## 2. Methods

### 2.1. Search Strategy and Study Selection

A search was performed for peer reviewed publications to identify guidelines specifically related to hepatitis B vaccination in preterm infants. The search strategy is outlined in Table 1. Relevant peer reviewed articles and guidelines published between 1 January 2002 and 12 July 2022 were found by searching the Ovid MEDLINE and Ovid Embase databases. We also searched bibliographies of the studies we identified and conducted targeted internet searches using conventional search engines (including Google Scholar) to find further relevant sources. The WHO database on hepatitis B vaccination schedules was used to cross-reference our search regarding current birth dose practice at country level [9]. We included all papers that were either published in English or for which English translations were available and limited the results to only include human studies.

### 2.2. Inclusion Criteria

The inclusion criteria were articles and guidelines that discussed the timing of the first dose of the hepatitis B vaccine in preterm infants born to HBsAg-negative mothers. Eligible sources were government and national immunisation program guidelines, and studies that referenced relevant national guidelines. Guidelines were only included if they specifically discussed preterm infants.

### 2.3. Exclusion Criteria

Studies were excluded if they met any of the following criteria: included animal subjects or published prior to 1 January 2002.

### 2.4. Systematic Review

The search results from all databases were imported to the Covidence systematic review software (Veritas Health Innovation, Melbourne, Australia), where duplicate articles were automatically removed. Two authors independently screened all articles based on their title and abstract to determine their eligibility, based on the inclusion and exclusion criteria. Findings from the relevant guidelines were then analysed and presented through narrative synthesis.

## 3. Results

**Figure 1:**
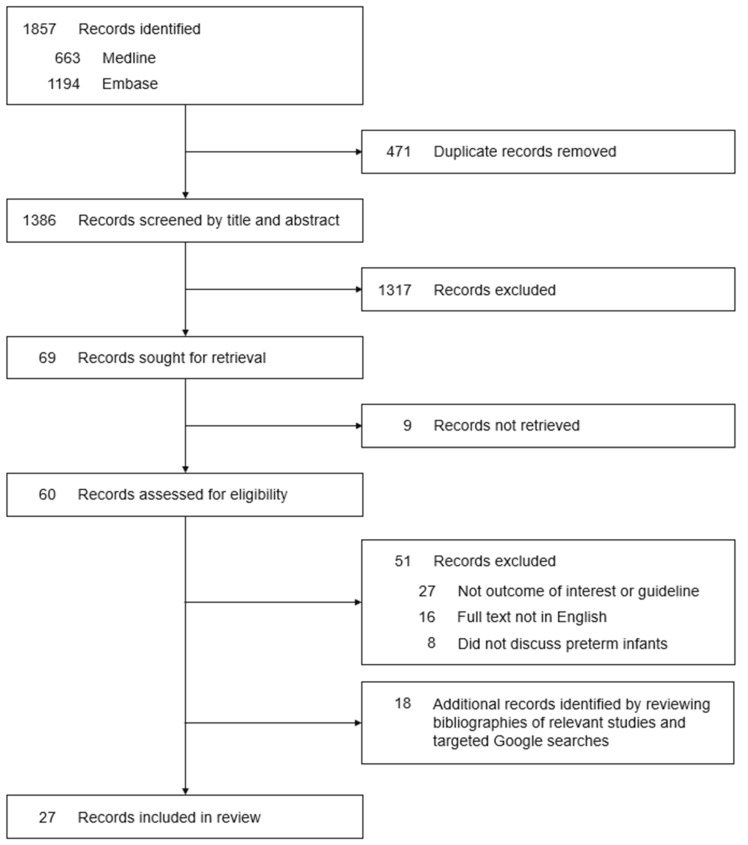
PRISMA flow chart of search results.

Our search strategy through Ovid MEDLINE and Ovid Embase identified a total of 1386 papers, of which 471 duplicate records had been removed. A total of 1317 articles were excluded after title and abstract screening because they did not discuss hepatitis B vaccinations in infants, and we were unable to access the full text of nine studies. A further 51 studies were excluded due to the following reasons: not outcome of interest or guideline (27), full text not in English (16) or did not discuss preterm infants (8). An additional 18 sources were identified by reviewing the bibliographies of relevant articles and by conducting targeted internet searches. In total, 27 papers and guidelines were eligible for inclusion in this review (Figure 1). These 27 documents pertain to guidelines that impact a population of 4.1 billion people, i.e., to approximately half of the world’s current total population.

Our results show that, despite WHO’s recommendation for all infants to receive their first dose of the hepatitis B vaccine as soon as possible after birth [5], guidelines vary between countries such as Australia, China, India, the United Kingdom (UK) and the United States of America (USA). In Canada and Macau, hepatitis B vaccination guidelines even varied within the country/region. In total, we identified 15 countries and regions for which relevant guidelines existed and were available to view online and in English. The guidelines we identified are summarised in Table 2, grouped according to their respective country or region. The most common reason for not listing a country/region was that we were unable to identify guidelines that specifically discussed the timing of the first dose of the hepatitis B vaccine in preterm infants.

### 3.1. Nationwide Birth Dose

Of the 15 countries/regions for which we were able to identify relevant guidelines, two (13.3% of countries/regions, 16.8% of the overall population affected by the guidelines included in this review) have guidelines stating that all infants, including preterm infants, should receive their first dose of the hepatitis B vaccine within 24 h of birth. These are Australia [10,11,12] and Latin America and the Caribbean [23]; note that Latin America and the Caribbean share the same guideline. Furthermore, these regions recommend an additional booster dose for particularly vulnerable preterm infants, defined in Australia as a birth weight < 2000 g or being born at a gestational age of < 32 weeks, and in Latin America and the Caribbean as a birth weight < 2000 g. Australian guidelines also discuss measuring hepatitis B surface antibody (HBsAb) titres in these vulnerable infants, with practitioners having the option to either measure antibody levels at seven months of age and provide the booster dose at 12 months of age if the titre is <10 mIU/mL, or to give the booster dose at 12 months of age regardless of antibody titres [11].

### 3.2. Nationwide Birth Dose Subject to Birth Weight

Six countries (40.0% of countries/regions, 77.9% of the overall population) have national guidelines that recommend a birth dose of the hepatitis B vaccine subject to the infant having reached a particular birth weight. These countries are China [15], India [17,18,19], Israel [22], Portugal [28], Taiwan [29] and the United States of America (USA) [31,32,33,34,35,36]. In China, India, Israel, Portugal and the USA, the birth weight threshold is 2000 g, whilst in Taiwan it is 2000–2200 g.

Each of these six countries outlines similar recommendations regarding when infants born below the birth weight threshold can receive their first dose of the hepatitis B vaccine. In general, these guidelines consist of three main criteria: the infant reaching a predefined weight or age, or being discharged from hospital. In China, infants with a birth weight of less than 2000 g are recommended to receive their first dose of the hepatitis B vaccine once their weight reaches ≥ 2000 g or just before discharge from hospital [15], whilst in Taiwan, guidelines recommend the first dose to be given once infants reach a weight of 2000–2200 g [29]. Indian guidelines state that the first dose should be given once the infant reaches 30 days of age and is medically stable [19], and in Portugal, preterm infants receive the first dose either at 2000 g or at one month of age, whichever occurs first [28]. Guidelines from the USA state that preterm infants with a birth weight of less than 2000 g should receive their first dose of the hepatitis B vaccine at either one month of age or at hospital discharge, if being discharged with a weight < 2000 g but are medically stable and consistently gaining weight [31,32,33,34,35,36]. The threshold in Israeli guidelines is similar and recommends infants to receive the first dose when they reach either 2000 g, one month of age or at hospital discharge, whichever occurs first [22].

### 3.3. Nationwide No Universal Birth Dose

Of the 15 countries/regions identified, five (33.3% of countries/regions, 4.4% of the overall population) have nationwide guidelines which do not include a universal birth dose. These countries instead recommend the first dose of the hepatitis B vaccine to be given during the first six to nine weeks of life to all infants, including preterm infants, as part of the routine hepatitis B vaccination series. These countries are Germany [16], Ireland [20,21], the Netherlands [25,26], New Zealand [27], and the United Kingdom (UK) [30]. The country with the earliest hepatitis B vaccination of these five is New Zealand, which recommends the first dose at six weeks of age for all infants, including medically stable preterm infants [27]. Dutch guidelines recommend practitioners to provide the first dose between six to nine weeks of age [25,26], whilst guidelines from Germany, Ireland and the UK state that all infants should receive their first dose of the hepatitis B vaccine at the age of two months [16,20,21] or eight weeks [30]. Of note, each of these countries recommend the first dose of the hepatitis B vaccine to be provided as part of a hexavalent combination vaccine (DTaP-IPV-Hib-HepB), providing protection against diphtheria, tetanus, pertussis, poliomyelitis, *Haemophilus influenzae* type b and hepatitis B [37].

### 3.4. Countries/Regions with Varying Guidelines

Of the relevant sources identified, our data indicated that two (13.3%/1.0%) countries/regions have guidelines regarding timing of the first dose of the hepatitis B vaccination in preterm infants that vary between jurisdictions [13,14] or hospitals [24]. The Government of Canada’s guidelines recommend that in provinces where infants routinely receive a birth dose of the hepatitis B vaccine, the first dose should be delayed in infants with a birth weight of less than 2000 g until they weigh 2000 g or at hospital discharge, whichever occurs first [13,14]. However, our search results did not identify guidelines specific to any Canadian jurisdictions. In Macau, guidelines at the Centro Hospitalar Conde de Sao Januario Hospital recommend all infants, including preterm infants, to receive the first dose of the hepatitis B vaccine at birth [24]. Additionally, preterm infants with a birth weight of less than 2000 g are recommended to have an additional booster dose during their second month of life. We were unable to identify guidelines from other hospitals in Macau regarding the timing of the first dose of the hepatitis B vaccination.

**Figure 2:**
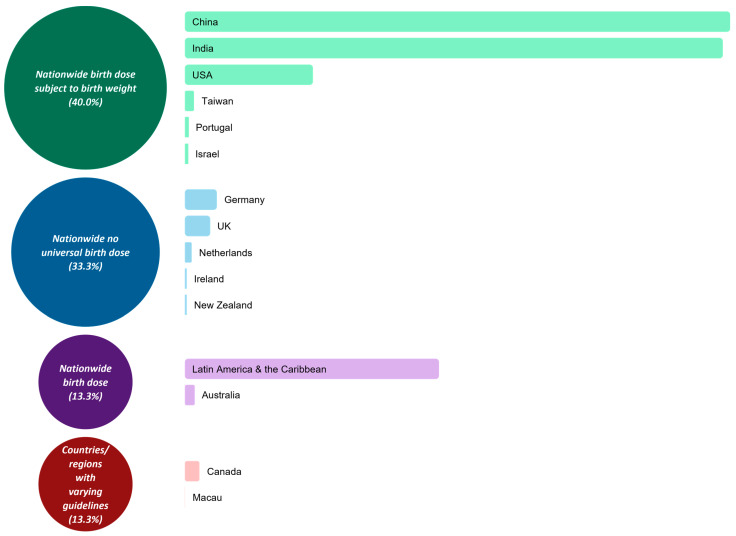
Summary of guidelines surrounding timing of the first dose of the hepatitis B vaccine in preterm infants born to HBsAg-negative mothers and countries/regions within each category. Area of circles is proportional to the number of countries/regions in each category, and the area of the rectangles is proportional to the population affected by the guideline. Total population of listed countries is 4.08 billion people [38].

The types of guidelines surrounding the timing of the first dose of the hepatitis B vaccine in preterm infants are summarised in Figure 2.

## 4. Discussion

Evidence from a recent longitudinal study in preterm infants and accompanying animal data revealed that type 2-polarised inflammation underpins the neonatal chronic lung disease, bronchopulmonary dysplasia (BPD) [8]. This type 2-polarisation was augmented if the hepatitis B vaccine was administered immediately after birth to the preterm infants enrolled in this study, but not if administration was delayed to approximately day seven of life (e.g., odds ratio (OR) 10.8 for a dose of the hepatitis B vaccine within the first 24 h of life vs. no vaccine within this period, 95% confidence interval (CI) 2.9–40, *p* = 0.0004; odds ratios were similar for the same comparison at weeks 1 and 2 of life, e.g., OR 28.5, 95% CI 3.0–273, *p* = 0.004) [8]. Notably, all of these infants were born to HBsAg-negative mothers. This finding raised the concern that the birth dose of the hepatitis B vaccine may be associated with an unfavourable immune polarisation in preterm infants and therefore may constitute a risk of harm in this population. Hence, preterm infants born to mothers not infected with hepatitis B might benefit from a delay of their first hepatitis B vaccine dose. This information raised questions about the best time to vaccinate these infants—which is the reason we conducted this review. However, we observed considerable variability in current international guidelines between countries and even within countries regarding this question, in both the timing of the first vaccination and doses used.

This variability in recommendations is at least in part justified by variations in hepatitis B prevalence and transmission rates, and therefore cost-effectiveness of different vaccination schedules. For example, hepatitis B vaccination for infants in the UK was not introduced until 2017, when the combination vaccine became available, for HBs-Ag negative mothers due to the low prevalence and low rates of vertical transmission [39] of hepatitis B in this country. It was determined that hepatitis B vaccination would only be cost-effective when a combination vaccine rather than a monovalent hepatitis B vaccine was used [40]. Since in infants < 6 weeks old only the monovalent preparation of the hepatitis B vaccine is approved [32], these infants had not been recommended to receive a birth dose.

In contrast, Australia and Latin America (13% of all regions, 17% of the overall population) have guidelines recommending a nationwide birth dose of the hepatitis B vaccine for all infants. The Australian Department of Health recommends that premature infants should receive vaccines in line with the recommended scheduling at chronological age without prematurity correction, provided they are medically stable with no contraindications to vaccination.

Administration of a birth dose reduces the risk of hepatitis B transmission in infants by up to 90% and is also associated with high seroprotective levels of > 95% especially when a four-dose schedule is used [41,42]. In preterm infants, apnoea, bradycardia and desaturations may be more likely to occur following immunisations [43,44], but these reactions can be safely mitigated with close monitoring and do not usually require intervention other than slight escalation of respiratory support. Beyond such early reactions, no other risk of harm has been demonstrated for preterm infants thus far; however, there is limited evidence in this field, particularly regarding vaccination timing.

A further 33% of countries/regions (4% of the overall population) had guidelines recommending no universal birth dose and instead the first dose at six to nine weeks of chronological age. One of the major benefits of later vaccination is that the hepatitis B vaccine can be provided as part of a combination vaccine to protect against other childhood illnesses that is only available to infants ≥ 6 weeks old [32]. Compared to monovalent vaccines, combined vaccination reduces the number of injections for the infant [45], increases vaccine coverage [46], is more convenient for families as it requires less clinic visits [47], and most commonly is more cost-effective [40].

The most common type of guideline (40%, 78% of the overall population) recommended a nationwide birth dose of the hepatitis B vaccine subject to infants being born with a birth weight of approximately 2000 g. These guidelines reflect existing literature, which shows high seroprotective levels of > 90% in preterm infants when the first dose of the hepatitis B vaccine is delayed until a weight of 2000 g is reached postnatally [48,49,50]. For example, in one study of preterm infants which compared those that received their first hepatitis B vaccine dose once they reached a weight of 1000 g vs. 2000 g found that 79% and 91% of infants, respectively, achieved seroprotective titres against hepatitis B [49].

The main limitations of our study were that we were only able to include countries with guidelines published online and in English and those that had specific recommendations for preterm infants. Compiled country-level data may not include details about preterm-specific guidance and not all individual country guidelines were reviewed. This circumstance limited our ability to describe patterns in timing of the first dose of the hepatitis B vaccination in preterm infants more widely. Where possible, dosing regimens of the hepatitis B vaccines were researched and outlined in Table 2 according to the guidelines of the regions and countries. However, there was wide variability in the level of information available on specific drugs licensed for use and on the specific dosing recommendations within the text of each guideline. Further, parts of our data for some countries and regions were extracted from articles which cited relevant guidelines rather than directly from published guidelines. As a result, it was difficult to determine the recency of the information we retrieved. When interpreting these results, it is also important to consider the differences between published guidelines and the actual implementation of these recommendations, as many barriers to timely vaccination of preterm infants have been identified, including concerns regarding the safety of the vaccine [51,52].

Future research should focus on weighing the risks and benefits of different approaches regarding timing of the first dose of the hepatitis B vaccine in preterm infants, including comparing the immunogenicity and safety of different schedules.

## 5. Conclusions

Guidelines relating to the timing of the first dose of the hepatitis B vaccine in preterm infants born to HBsAg-negative mothers show considerable variation between countries and regions. The guidelines most commonly adopted by countries/regions include a birth dose that is subject to infants being born with a birth weight of at least 2000–2200 g, or no universal birth dose and instead the first dose at six to nine weeks of age for all infants. The large variability between guidelines is at least in part justified by factors specific to countries and regions, such as the prevalence of hepatitis B infection. However, we argue that new safety concerns regarding the birth dose in preterm infants should be considered going forward, particularly in regions in which have comprehensive maternal hepatitis B screening programs.

## Figures and Tables

**Table 1:** Search strategy—search items and MEDLINE medical subject heading (MeSH) terms used.

Concept	Search Items	MeSH Terms
Vaccination	vaccin * OR immunis * OR immuniz * OR inoculat *	“vaccination”, “vaccines”, “viral vaccines” “immunization”, “immunization programs”, “vaccines, DNA”
Preterm	preterm OR prematur *	“infant, premature”, “premature birth”
Infant	infant OR neonat * OR baby OR babies	“infant, extremely premature”, “infant, premature, diseases”
Timing	timing OR time OR timetable OR schedule OR program * OR plan OR guideline * OR strateg * OR procedur *	“practice guideline”

**Table 2:** Summary of guidelines on timing of the first dose of the hepatitis B vaccine in preterm infants born to HBsAg-negative mothers. Footnotes in this table refer to vaccination details.

Country/Region	References	Guideline
Australia	Australian Immunisation Handbook (2021) [10] Chaudhari (2021) [11] Australian Immunisation Handbook (2020) [12]	Preterm infants receive the first dose of Engerix-B ^1^ or H-B-Vax II ^2^ within 24 h of birth, if medically stable. Infants with a birth weight < 2000 g or gestation < 32 weeks receive an additional booster dose at 12 months of age.
Canada	Government of Canada (2021) [13] Government of Canada (2020) [14]	In jurisdictions where guidelines recommend infants to receive the first dose of Engerix-B ^1^ or Recombivax ^3^ at birth, this recommendation includes a delay of this first dose in infants with a birth weight < 2000 g until the infant reaches 2000 g or at hospital discharge, whichever occurs first.
China	Zhou et al. (2020) [15]	Preterm infants with a birth weight ≥ 2000 g receive the first dose at birth ^4^, if medically stable. Preterm infants who are not medically stable should receive appropriate management and be given the first dose 1 week after becoming medically stable. Infants with a birth weight < 2000 g receive the first dose ^4^ when the infant reaches 2000 g or at hospital discharge.
Germany	Robert Koch Institute (2022) [16]	Preterm infants receive the first dose ^5^ at 2 months of age as part of a combination vaccine (DTaP-IPA-Hib-HepB).
India	Ministry of Health & Family Welfare, Government of India (2018) [17] Ministry of Health & Family Welfare, Government of India (2017) [18] Dutta (2014) [19]	Preterm infants with a birth weight ≥ 2000 g receive the first dose ^6^ within 24 h of birth. Infants with a birth weight < 2000 g receive the first dose ^6^ at 30 days of age and when medically stable.
Ireland	Health Service Executive (2022) [20] Health Service Executive (2022) [21]	Preterm infants receive the first dose ^7^ at 2 months of age as part of a hexavalent vaccine.
Israel	Ministry of Health, State of Israel (2022) [22]	Preterm infants with a birth weight ≥ 2000 g receive the first dose shortly after birth ^8^. Preterm infants with a birth weight < 2000 g receive the first dose ^8^ when the infant reaches 2000 g or at hospital discharge or at 1 month of age, whichever occurs first. Infants discharged before reaching 1 month of age or 2000 g can receive the first dose ^8^ if medically stable and consistently gaining weight.
Latin America and the Caribbean	Pan American Health Organization (2017) [23]	Preterm infants receive the first dose ^9^ as soon as possible after birth, preferably within 24 h. Preterm infants with a birth weight < 2000 g require an additional booster dose.
Macau	Centro Hospitalar Conde de Sao Januario hospital guidelines as per Choi et al. (2019) [24]	Preterm infants receive the first dose at birth ^10^. Preterm infants with a birth weight < 2000 g receive a booster dose during their 2nd month of life, in addition to the routine schedule.
Netherlands	Rouers et al. (2019) [25] Scheepers et al. (2017) [26]	Preterm infants receive the first dose ^11^ between 6 and 9 weeks of age as part of a combination vaccine (DTaP-IPV-Hib-HepB).
New Zealand	Ministry of Health, New Zealand Government (2020) [27]	Preterm infants receive the first dose ^7^ at 6 weeks as part of a hexavalent vaccine, if medically stable.
Portugal	SNS24 (2022) [28]	Preterm infants with a birth weight ≥ 2000 g receive the first dose at birth ^12^. Preterm infants with a birth weight < 2000 g receive the first dose ^12^ when the infant reaches 2000 g or at 1 month of age, whichever occurs first.
Taiwan	Chen et al. (2014) [29]	Preterm infants with a birth weight ≥ 2000–2200 g receive the first dose at birth ^13^. Preterm infants with a birth weight < 2000–2200 g receive the first dose ^13^ when the infant reaches 2000–2200 g.
United Kingdom	UK Health Security Agency (2013) [30]	Preterm infants receive the first dose ^7^ at 8 weeks of age as part of a hexavalent vaccine.
United States of America	Centers for Disease Control and Prevention (2021) [31] AAP Committee on Infectious Diseases (2018) [32] Phillips et al. (2013) [33] Advisory Committee on Immunization Practices (2011) [34] AAP Committee on Infectious Diseases (2003) [35] Advisory Committee on Immunization Practices and American Academy of Family Physicians (2002) [36]	Preterm infants with a birth weight ≥ 2000 g receive the first dose within 24 h of birth ^1,3^. Preterm infants with a birth weight < 2000 g receive the first dose ^1,3^ when the infant reaches 1 month of age or at hospital discharge, whichever occurs first.

^1^ Engerix-B Paediatric (1.0 mL = 10 µg hepatitis B surface antigen protein); ^2^ H-B-Vax II (0.5 mL = 5 µg Hepatitis B surface antigen protein); ^3^ Recombivax HB-Paediatric (0.5 mL = 5 µg Hepatitis B surface antigen protein); ^4^ Recommended dose 0.5 mL = 5 µg; no specific vaccine details provided; ^5^ Infanrix Hexa, Hexyon, Vaxelis; guideline does not specify dosage; ^6^ Recommended dose 0.5 mL of a hepatitis B vaccine; no hepatitis B surface antigen protein dose or drug name specified; ^7^ Infanrix Hexa (DTaP-HepB-IPV-Hib); 0.5 mL = 10 µg hepatitis B surface antigen protein; ^8^ Guideline does not specify vaccine drug name or dosage; ^9^ Guideline mentions the replacement of the plasma-derived hepatitis B vaccine with a recombinant hepatitis B vaccine. Recommended dosage of 5 µg in 0.5 mL suspension, guideline does not specify vaccine drug name; ^10^ Paper does not mention vaccine type or dosage recommendations; ^11^ DTaP-IPV-Hib-HBV vaccine, but no dose or vaccine drug name specified; ^12^ Recommended dose 0.5 mL (5 or 10 µg antigen depending on the manufacturer), drug name not specified; ^13^ Recombinant HBV vaccine, Engerix-B (20 μg per 1 mL) or H-B-Vax II (5 μg per 0.5 mL).

## Data Availability

Detailed search data available from authors on request.
